# The Stable Door and the Stolen Steed

**Published:** 1892-02-06

**Authors:** 


*For Hospitals, Asylums, Infirmaries, Dispensaries, Medical Practitioners, Students, Nurses, and the Charitable Public.*

Vol. XI.—No. 280.] [Feb. 6, 1892.

### THE STABLE DOOR AND THE STOLEN STEED.

It cannot be denied that people who are not doctors have some feeling of dissatisfaction, not to say resentment, against the hospitals and the members of the medical profession in connection with the present epidemic. Before the issue of the circular of the Local Government Board the daily newspapers protested because nothing was done; after the circular had been studied they protested with still more energy that what had been done was not worth the doing. There is not an intelligent medical man in the kingdom but will sympathise with the constructor of the proclamation of the Local Government Board. Medical men are taught to be, above all things, correct in their knowledge. They know that whatever they publish will be scanned by a thousand eager and critical eyes, and that judgment of a character which will permanently influence their career will be silently but sternly passed upon their work by a court whose verdict is hardly ever revoked.

The man who thoroughly understands the spirit of modern medicine publishes with fear and trembling. It is true that the number of medical books and publications becomes greater every year. But those books are for the most part written by the fools who "rush in where angels fear to tread." The writers of them have no conception of the risks they run. They do not dream that the book which they send forth into the little world of medicine will be the one circumstance in their lives that will sink them to depths of scornful contempt in the estimation of the more intelligent members of their profession from which they can never rise again. One need not wonder that the really cultured medical men who are learned enough to "know what they don't know," shrink with almost mortal terror from throwing themselves and their work into a seething critical cauldron of this character. Dr. Thorne Thorne undoubtedly has the sympathy of most his enlightened confrères in both the slowness and the timidity of his action. It is quite plain that he had very little valuable knowledge to impart; and it is equally evident that he has not been able to persuade himself to make a pretence of knowing what he did not know, or of offering with a show of high authority the mere commonplaces of every day knowledge and experience.

But the man in the street is not satisfied with this kind of thing. He is not a specialist, and he cannot understand the temper of mind which controls the action of the specialist. It seems to him that the last effect of high study and culture is to make almost every man sit down and fold his arms in the midst of the greatest of the world's troubles with a helpless and hopeless *non possumus*. What is the use of the medical profession, he scornfully asks, if it can do nothing in the midst of a dangerous epidemic but tell us to go to bed and take care of ourselves? After three seasons of the influenza scourge we do not require an army of expensively-educated men to tell us that. The members of the general public expect something to be done, and to be done with intelligence and promptitude. The cultured members of the medical profession are quite willing to do something if they only knew what. But they are so hampered by a critical environment of minute and formal correctness that they hardly dare stir hand or foot lest they should immediately attract the arrow of critical scorn in their own direction.

This, we submit, is an unworthy attitude for the leaders of the medical profession to take up. They know well the general nature of influenza, at least in the effects it produces; and they know also that certain very commonplace and obvious causes directly tend to encourage the spread of the disease. There are scores of physicians in London who could make excellent summaries of predisposing and exciting causes, of the nature and progress of the symptoms, of rational methods of cure, and of equally rational and effective methods of prevention. The helpless thousands of our population present an appeal to medical science which is both urgent and pathetic. They say in effect, If you do not know all about the influenza, tell us what you do know; tell us even what you think; and tell us plainly, so that we may understand at least what you yourselves understand. You seem to us to be quite apathetic and indifferent to our anxieties and sufferings. You make light of what appears to everybody but yourselves a dangerous and fatal plague.

It is a pity that medical men as a body separate themselves so absolutely and completely from the public in its corporate capacity. Individual medical men move naturally and freely among their own patients, and spread a great deal of valuable knowledge from house to house and from day to day. But medicine as a science, and its members as a profession, hold themselves as entirely aloof from the great body of their fellow citizens as if they lived in another planet. The medical mind in London has given itself up, almost without an attempt at resistance, to the bondage of a conventional professionalism. There is no logic in such a situation; and most certainly there is no humanity. If it be right for the individual doctor, not only to cure those who may be attacked by the prevailing scourge, but to isolate the sick, and to teach the households of his patients every method he can devise for the checking of the spread of the disease, how much more largely right would it be if the whole body of the medical profession, acting in concert like a well directed army, could make a great national effort to deliver their fellow citizens from the fear and danger of an alarming and fatal disease!

The profession of healing, in an epidemic like the present, is not on parade, it is in the thick of a conflict with death. The army that has to meet an opposing army in mortal struggle has no thought to waste upon little accidents of personal appearance and parade-ground nicety. It is there to fight; and fight it must, or be routed and destroyed. Let no self-pleased physician think himself and his order above the criticism of the common and strenuous world. The members of the medical profession are but a part of the common world; they are the servants of the world. If the world finds them unprofitable servants it will know how to punish them. The doctor who is loyal to his profession reads with alarm the contemptuous comments of the lay press upon the helplessness of the defenders and conservators of the public health. Of course, the scribes who teach the public in the lay press cannot look at these questions from the doctor's standpoint, and herein they are at a serious disadvantage. But the doctor can look at them from the standpoint of the public if he will, and it is both his duty and his interest so to do. If from such an advantageous position as this he falls short of what a large and generous humanity should prompt him to do, he invites the disapproval and angry contempt of all those who have found him a defender who failed of his duty at the critical hour of need.

There are those who say that, since we have had two epidemic seasons before the present one, and since history has taught us to expect three successive seasons with some certainty, we should have been prepared for the present winter outbreak should have had our methods of prevention matured and our plans of treatment agreed upon; and should so have given the public that assurance of readiness and capacity which it had a reasonable right to expect. Instead of that, it is said that the present winter has found us as ignorant, as helpless, as divergent in opinion, and as entirely chaotic in every sense as we were found to be in the two previous winters. This is not, perhaps, altogether just; but there is enough of justice in it to make us wish that a plan of campaign had been agreed upon, and that the public had found us as ready to render all the needed service as it has found us unprepared.

There are some who remind us, in connection with the proposed Enquiry Commission, that it is not wise men who "lock the stable door when the steed is stolen," but men to whom a very different epithet should be applied. This criticism, too, it must be admitted, has at least an appearance of justice. It is a pity, a very great pity, both for the public and the medical profession, that it should be so. Why was not the Commission suggested at the beginning of the second season of the outbreak instead of at the end of the third? If history should repeat itself in the present instance, as it has so often done in the past, the current epidemic will be over before the investigators have well begun to take evidence; and it is probable that no more will be heard of influenza until the members of the Enquiry Commission have long been sleeping the sleep that knows no waking. The medical profession certainly cannot congratulate itself on the way in which it has managed the influenza epidemic; but if the sharp criticism which has been so universally evoked should bring us to a more humanly rational and less narrowly professional state of mind, the epidemic, although it may have been a great, will not have been altogether an unmixed evil.

